# Promoting Pro-environmental Beliefs and Behaviour: Choose-Your-Own Story Futuristic Climate Game

**DOI:** 10.1371/journal.pone.0317773

**Published:** 2025-03-31

**Authors:** Lala Muradova, Edana Beauvais

**Affiliations:** 1 Department of Politics and International Relations, University of Southampton, Southampton, United Kingdom; 2 Department of Political Science, Simon Fraser University, Burnaby, BC, Canada; University of Valencia: Universitat de Valencia, SPAIN

## Abstract

How can we address climate scepticism and increase public support for ambitious pro-environmental policies? This study investigates the potential of future-oriented perspective taking, using an innovative and futuristic choose-your-own-adventure narrative game. This cutting-edge intervention involves living in the life of a future self and making choices related to hypothetical climate crises. The choose-your-own-adventure game was integrated into online survey experiments in the United Kingdom (*N* = 1,738) and the United States (*N* =  1,290). We found that participation in the game elicited strong emotional responses in individuals, making them more empathetic, but also more hopeless and sad. Imagining their future self during the climate game enhanced people’s willingness to engage in future discussions about climate change among the UK respondents. Yet, the intervention did little to transform people’s pro-environmental beliefs, policy support, or willingness to sign a climate petition. Causal mediation analyses reveal that these null effects hide important direct and indirect effects. Empathic concern mediates significant positive indirect effect of climate game on people’s pro-environmental beliefs, but negative indirect effect on willingness to sign the climate petition. Empathy seems to shape environmental beliefs and behaviours in diverse ways, highlighting the complex and nuanced relationship between them. These findings offer important implications for recent research on the role of emotions in climate change communication, environmental psychology, and policymaking. We also present a unique approach to fostering empathy for the environment and future generations through an engaging choose-your-own-adventure game.

## Introduction

Climate change represents the most pressing global challenge of the 21st century. Despite scientific consensus on the anthropogenic cause of climate change, a non-trivial segment of the world’s population still holds skeptical beliefs about climate change, opposes ambitious policies to address climate change, and is reluctant to adopt pro-environmental behaviors [1–4]. How can we increase concern for the impacts of climate change, stimulate support for policies designed to address climate change, and motivate citizens’ political action on climate change? Research shows that pro-environmental policy preferences, and behaviours are influenced by individual socio-demographic (e.g., age, gender) and psychological characteristics [5,6], contextual factors, such as social norms [7–9], institutional design features of policies [10], and the framing of climate change communications [1,11].

Social science experiments reveal that promoting pro-environmental beliefs and behaviours is not easy. Rare are studies demonstrating homogenous and substantially important effects of an intervention on climate beliefs and behaviour [12,13]. Recent behavioural research highlights the potential of emotion-driven interventions to transform pro-environmental beliefs and actions [14–16]. However, empirical evidence remains inconsistent. For instance, fear has been shown to have positive, mixed, or negligible effects on individuals’ attitudes and behaviours [17–20]. The evidence on the effectiveness of hope-inducing interventions is also mixed [21].

The potential of other emotions, such as *empathy,* is largely understudied. One of the recurring findings in social and political psychology is that inducing empathy via perspective-taking interventions can help people understand the world from different perspectives, motivate them feel empathy toward different others and the environment, and catalyze important attitudinal and behavioral changes [22–26]. These studies raise the possibility that empathic perspective taking interventions could motivate more pro-environmental beliefs and behavior in individuals. Such an intervention is reported here. We design a first-person, choose-your-own-adventure narrative game that involves living a few days in the life of a player’s future self and navigating the consequences of climate disasters, flooding, wildfires, drought, erosion, and food shortages, in the year 2121. The player becomes the story’s main character, making key decisions to address the personal consequences of future climate disasters.

We bring together several strands of literature in designing our intervention. First, our design incorporates future scenarios. Research finds that future scenarios can be effective in engaging local communities in local climate change adaptation measures [27]. Our scenarios are based on scientific predictions about the UK and US climate in a hundred years’ time. Second, the intervention aims to elicit empathic perspective taking in individuals, by motivating them to see and imagine natural threats and risks related to climate change. Previous literature shows that empathic concern mediates the effect of perspective taking interventions on a range of attitudes [28]. Third, we designed our game in the choose-your-own-adventure style, which harnesses the power of experiential learning and imagination (see [29] for other types of climate games), decreases psychological distance between people and the environment, and increases emotional engagement [30]. Previous research finds that direct personal experience of climate-related hazards is positively associated with people’s increased risk perceptions about climate change [31–33], and imaginatively experiencing climate disasters could be a powerful tool for enhancing people’s understanding of the implications of climate change for themselves and for others. Lastly, our intervention differs from other instructional interventions that elicit empathy, which are commonly used in behavioural sciences, due to its more realistic setting and engaging nature.

### Aims and hypotheses

The primary aim of this study is to examine the research question (RQ1) of whether adopting the perspective of one’s future self during hypothetical climate crises can increase individuals’ climate change-related beliefs, pro-environmental policy support, and behaviour. Our first set of hypotheses (H1) predict that taking the perspective of a future self via a climate choose-your-own-adventure game will promote pro-environmental (H1a) beliefs, (H1b) policy support, and (H1c) behaviour. The second research question (RQ2) explores the nature and intensity of emotions respondents experience while playing the climate game. More specifically, we are interested in whether the choose-your-own-adventure climate game enhances individuals’ empathetic concern (H2a) and if empathy mediates the effect of the game on climate attitudes, policy preferences, and actions (H2b).

## Methods

### Intervention

**Fig 1:**
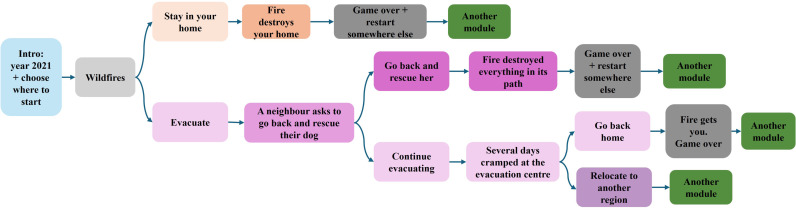
A part of the climate choose-your-own-adventure narrative map (Fires Module).

**Fig 2:**
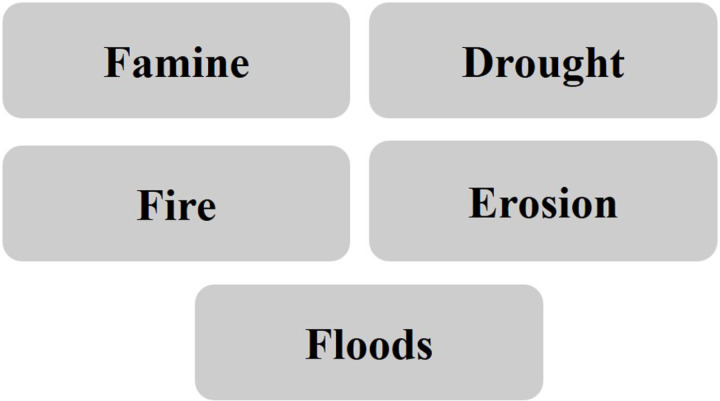
The list of scenario modules.

The intervention is a choose-your-own-adventure game that involves being the main character of the story and navigating the consequences of a series of climate disasters — flooding, wildfires, drought, erosion and food shortages (see Fig 1 for a sketch). Each participant plays the game one time. While playing the online game, the participant is asked to make several important decisions within each climate crisis module. In designing five different climate risk scenarios (Fig 2), we drew on the UK government’s UK Climate Change Risk Assessment (Study 1, UK), and the Environmental Protection Agency’s (EPA’s) Climate Change Impacts by State document (Study 2, US). We linked each risk scenario with relevant regions in the UK, and states in the US, also using government reports and recent news articles to determine which regions/states would be most realistically and saliently associated with each climate risk (see Table 1).

**Table 1:** Regions/states and the related climate crisis modules.

Region/State	Climate Risk
UK	
Midlands (East or West); or Southwest of England; or Northern Ireland	Drought Module
East of England, or Wales	Erosion Module
Northwest England or Southeast	Fire Module
London	Famine (Food Scarcity) Module
Scotland, or Northeast, or Yorkshire, and Humberside	Flooding Module
US	
Kansas, Nebraska, Kentucky, Nevada, New Mexico, Oklahoma, Utah, Wyoming.	Drought Module
Alabama, Alaska, Delaware, Georgia, Hawaii, Louisiana, Maryland, Mississippi, New Jersey, New York, North Carolina, South Carolina, Texas, and Virginia.	Erosion Module
Arizona, California, Colorado, Idaho, Montana, Oregon, and Washington.	Fire Module
Connecticut, District of Columbia (DC), Massachusetts, and Rhode Island.	Famine (Food Scarcity) Module
Arkansas, Florida, Illinois, Indiana, Iowa, Main, Michigan, Minnesota, Missouri, North Dakota, Ohio, Pennsylvania, South Dakota, Tennessee, Vermont, West Virginia, and Wisconsin.	Flooding Module

**Fig 3:**
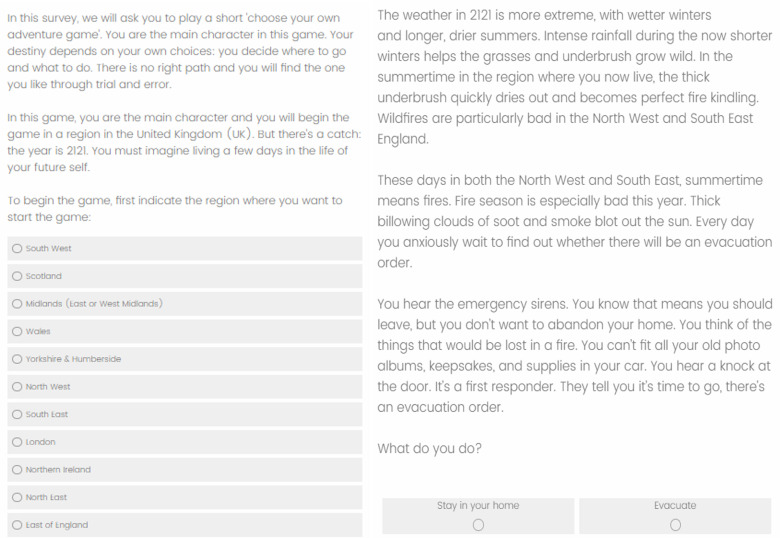
An example choose-your-own-adventure choice scenario (introduction +  fire module scenario) (UK version).

The climate game was designed by the authors of this manuscript, building on the genre of choose-your-own-adventure books, for example, the 1970s famous books of *The Cave of Time*, by Edward Packard, and *Journey under the sea*, by R.A. Montgomery, among others (see also Simonovits et al. 2018 for a choose-your-own-adventure style intervention about Roma minority in Hungary). It is a text-based narrative game (see Fig 3 and S1 Fig for an example). Respondents who were randomly assigned to play the climate game were told that they would experience life as their future self, in the year 2121 (UK), or year 2122 (US), and are asked in which region in the UK/which state in the US they would like to start the game.

After making a series of choices within the first relevant climate-risk module (conditional on their region/state choice), respondents were given the option to “move to another region of the UK/another state in the US” or else, eventually reaching a “game over,” at which time they “started over in another region”, and were randomly assigned to the next climate risk module. All participants in the treatment condition played their way through every climate-risk module (restarting their lives in different UK/US regions), regardless of where they start the game. Individuals played the full choose-your-own-adventure narrative game only once. The game lasted approximately 17–20 minutes.

### Ethics statement

The experimental procedure, and the surveys were approved by the Research Ethics Boards (REB) of the University of Leuven (KU Leuven), and Simon Fraser University (Canada). All participants provided written informed consent and were compensated for their participation. The studies were preregistered at aspredicted.org/tm56-5wsr.pdf; with the amendments being preregistered at https://aspredicted.org/mrgq-7jh9.pdf, and aspredicted.org/ytnr-mrkg.pdf. All replication materials (data and codes) are available at HarvardDataVerse https://doi.org/10.7910/DVN/T8ZDZS. See S1 File for the list of deviations from the preregistration plan.

### Study design

**Fig 4:**
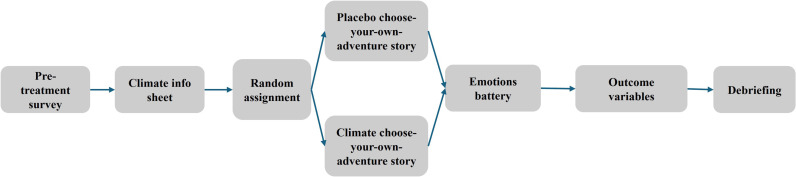
The Design of the Survey Experiments.

The choose-your-own-adventure intervention was embedded in a survey taken by individuals online at their convenience (Fig 4). We designed and fielded two nationally representative survey experiments in the United Kingdom (Study 1, *N* = 1,738) and United States (Study 2, *N* = 1,290).

After reading about the project and consenting to take part, individuals were asked to respond a set of questions about themselves, such as their age, gender and level of education, among others. We also measured respondents’ baseline party identity and political ideology. Further, all subjects read a half-page information about how climate change is leading to more extreme weather in different regions of their respective country (UK or US). Respondents were told that the question containing climate change information was designed to ensure they were paying attention. In fact, this was done to ensure all participants had the same informational background, thereby keeping learning about climate change constant across treatment conditions. In Study 2, we also included a placebo condition with no information on climate change. Participants were then randomly assigned to one of two (or three in the US context) experimental conditions: placebo or treatment. Respondents who were randomly assigned to the placebo condition played a choose-your-own-adventure fantasy game that was unrelated to climate change. In the placebo game, respondents took the role of an aspiring hero and navigated a series of adventures on the fictional Solinelia Island, for instance, by battling winged beasts that attacked the hero’s village. Respondents who made it to the end of the game discovered that their adventures on Solinelia Island may have been a dream.

Respondents who were assigned to the treatment condition were invited to play the climate change related choose-your-own-adventure game. We measured all respondents’ experienced emotions and our main outcome variables, after they completed their game (either the placebo or treatment game). To conclude the study, respondents were debriefed about the study objectives and thanked for their participation.

#### Samples (study 1 and study 2).

Study 1 was fielded between 19/02/2021 and 13/04/2021, among *N* = 1,738 UK individuals, over the age of 18. Approximately 53% of the sample identified as women, 4% as transgender or non-binary, and the remainder as men. In terms of education, 41% of respondents reported having post-secondary education, while only 1.2% had not completed secondary school. Politically, the sample included 37.7% who identified as Conservatives, 26.8% as Labour supporters, and 9.7% as Liberal Democrat supporters. The sample demonstrated significant regional diversity, for example, with 12.7% residing in London and 14.4% in the Southeast of the UK, while only 1.9% reported living in Northern Ireland (see S1 Table).

Study 2 was fielded between 28/06/2022 and 19/07/2022. The Study 2 sample consisted of *N* = 1,290 adult Americans, with a gender distribution of 52.6% identifying as female, 46.5% as male, and 0.85% as transgender, non-binary, or another gender identity. The participants’ ages ranged from 18 to 81 years, with an average age of 46.8. Among the 1,242 respondents who answered the partisanship question, 46.2% identified as Democrats, 28.6% as Republicans, and 25.2% as either independents or affiliated with another party. Some 30.6% of participants reported having at least a bachelor’s degree. The sample also demonstrated regional diversity, with respondents representing various geographic areas (S2 Table).

### Measurements

#### Dependent variables.

We are interested in three sets of outcome variables (reported in Table 2). The first measures people’s beliefs about climate change. It includes four different questions capturing public beliefs about the anthropogenic causes of climate change and its future impact at individual and generational levels. One of these question items was measured pre- and then post-treatment (see below). A second set focuses on individuals’ support for three different pro-environmental policies, all measured post-treatment. We also capture self-reported pro-environmental behavior with two questions. The first question captures individuals’ willingness to sign a climate petition, and the second their intention to discuss climate change with different others in the future. We measured the latter question also at the start of the survey, pre-treatment. The list of descriptive statistics (e.g., sample means and standard deviations) can be found in S3-S4 Tables.

**Table 2:** The List of Main Variables of Interest.

Experienced emotions	Please indicate the extent to which you felt the following emotions (if any) while participating in the choose-your-own-adventure game. The listed emotions are: sympathy, empathy, concern, compassion, fear, sadness, anger, disgust, surprise, happiness, grief, worry, indifference, guilt, helplessness	1 Not at all 2 A little 3 A moderate amount 4 A lot 5 A great deal
Climate change beliefs	You may have heard the idea that the world’s climate is changing due to increases in temperature over the past 100 years. What is your opinion on this? Do you think the world’s climate is changing?	1 Definitely not changing 2 Probably not changing 3 Might or might not be changing 4 Probably changing 5 Definitely changing
	If you think climate change is happening, do you think it is caused by natural processes, human activity, or both?	1 I don’t think climate change is happening. 2 Almost entirely by natural processes. 3 About equally by natural processes and human activity. 4 Almost entirely by human activity.
	How much do you think climate change will harm future generations of people?	*Reverse coded to mean:* 1 Not at all 2 A little 3 A moderate amount 4 A lot 5 A great deal
	How much do you think climate change will harm you personally?	*Reverse coded to mean:* 1 Not at all 2 A little 3 A moderate amount 4 A lot 5 A great deal
Pro-environmental policy support	To what extent are you in favour or against the following policies in the UK to reduce climate change? Increasing taxes on fossil fuels, such as oil, gas and coal	1 Strongly against 2 Somewhat against 3 Neither against nor in favour 4 Somewhat in favour 5 Strongly in favour 6 Don’t know
	Using public money to subsidise renewable energy such as wind and solar power	1 Strongly against 2 Somewhat against 3 Neither against nor in favour 4 Somewhat in favour 5 Strongly in favour 6 Don’t know
	A law banning the sale of the least energy efficient household appliances	1 Strongly against 2 Somewhat against 3 Neither against nor in favour 4 Somewhat in favour 5 Strongly in favour 6 Don’t know
Pro-environmental behaviours	Would you like to sign the ‘StopGlobalWarming’ online petition? StopGlobalWarming is a movement about creating change, as individuals, as a country, and as a global community. Join the 1,140,032 supporters of the Stop Global Warming Virtual March, and become part of the movement to demand our leaders freeze and reduce carbon dioxide emissions now. We are all contributors to climate change and we all need to be part of the solution.	*Reverse coded to mean:* 1 Don’t sign the petition. 2 Sign the petition.
	Going forward, how often do you intend to discuss climate change with others?	*Reverse coded to mean:* 1 Not at all 2 A little 3 A moderate amount 4 A lot 5 A great deal

#### Mediating variable(s).

We are also interested in the emotional consequences of climate game for individuals. After playing the game, participants were asked to report the intensity of the emotions they experienced. Self-reported experienced emotions were captured with a battery item (see Table 2), which included fifteen emotion words in the UK sample, and eleven emotion words in the US sample (sympathy, empathy, concern, compassion, fear, sadness, anger, happiness, worry, guilt and helplessness). In this study, we are primarily interested in empathic concern. Consistent with the previous literature [e.g., 23], we measure empathic concern with several empathy adjectives. Respondents were asked to indicate the extent to which they felt sympathetic, empathic, concerned, and moved when participating in the game. All four items in the battery loaded well in one factor, with high internal consistency (Cronbach’s alpha =  0.90). The items were summed to form an index measure, and the index was further rescaled to 0–1 to facilitate the interpretation and comparison of effects.

#### Manipulation check.

To examine whether the treatment was taken correctly by respondents in our sample, after the game, we asked respondents the following question: “Please indicate the extent to which you agree with the following statement: ‘when playing the choose-your-own adventure game, I imagined how my future self would feel and think’, with response categories being (1) strongly disagree, (2) disagree, (3) neither disagree nor agree, (4) agree, and (5) strongly agree.”

#### Analysis plan.

To investigate our research questions, and test the effects of our climate intervention, we conducted difference-in-means t-tests and OLS regression analyses. The main models reported in this paper are bivariate. For robustness, we rerun the models controlling for a set of pre-treatment covariates, specifically: age, gender, education, race, political party identity, and political ideology (left-right spectrum). To capture within-subject changes in intention to discuss climate change in the future, one of our behaviour measures, we run a paired sample t-test by treatment conditions. Lastly, to investigate the mediating effects of empathic concern on pro-environmental beliefs, policy support and climate action, we conducted causal mediation analyses [34]. For these analyses, we create two index variables by summing items that measure climate change beliefs and pro-environmental policy preferences. We analysed two pro-environmental behaviour measures (signing a petition and intention to discuss climate change) separately.

## Results

### Study 1 (UK)

Our manipulation check shows that respondents in the climate game were more likely to imagine their future (M = 3.85; SD = 0.93), than those in the placebo game condition (M = 3.26; SD = 1.10) and the difference (MD = 0.59; SD = 0.05) is statistically significant at two-tailed *p* < 0.01 significance level. Expressed in percentages, 72.4% of respondents in the treatment condition reported that they imagined their future self as opposed to only 49% of respondents in the placebo condition. This gives us an indication that the majority of respondents in the experimental treatment engaged in perspective taking.

**Fig 5:**
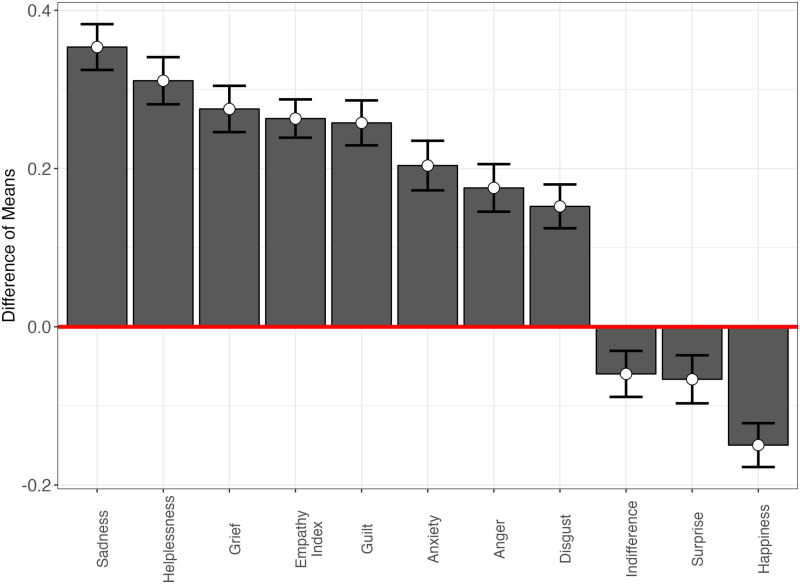
Mean differences in experienced emotions, by treatment condition (Study 1).

Furthermore, using the difference-in-means to recover the average treatment effect (ATE), we find that the choose-your-own-story climate game elicited more empathic concern (MD = 0.26; SE = 0.01; two-tailed p < 0.01) in individuals (Fig 5).

As Fig 5 shows, playing the game also increased sadness, helplessness, grief, guilt, anxiety, anger and disgust, while reducing indifference, surprise and happiness. In other words, the choose-your-own-adventure climate game emotionally engaged respondents, evoking a range of negative emotions alongside fostering increased empathy.

To investigate our first set of hypotheses, i.e., the effects of playing the climate game on pro-environmental beliefs, policy support and action, we conducted simple OLS regression analyses. The full results of the regression models are reported in Table 3. Contrary to our expectations, we find that playing the climate change game did not directly increase people’s belief that the world’s climate is changing due to increases in temperature over the past 100 years. Neither did it directly increase the public belief that climate change is caused by human activity, nor did it increase the belief that climate change will harm them personally or harm future generations. Paired t-test shows that playing the climate game had no within-subject effect on the belief that climate change will harm the future generations. We also found no direct effect of playing the climate game on support for climate policies, such as support for increasing taxes on fossil fuels, using public money to subsidise renewable energy, or the law banning the sale of the least energy efficient household appliances. Lastly, we found no direct effect of playing the game on pro-environmental behaviour, including willingness to sign a petition and willingness to talk about climate change in the future.

**Table 3:** The effects of climate game on pro-environmental beliefs, policy support and action.

	M1	M2	M3	M4	M5	M6	M7	M8	M9
Climate Game	−0.02	−0.01	0.07	−0.02	0.03	0.03	0.06	0.06	0.01
	(0.04)	(0.03)	(0.05)	(0.05)	(0.06)	(0.05)	(0.06)	(0.06)	(0.02)
_cons	4.31^***^	3.31^***^	2.76^***^	3.96^***^	2.50^***^	2.08^***^	2.22^***^	2.86^***^	1.48^***^
	(0.03)	(0.03)	(0.04)	(0.04)	(0.04)	(0.04)	(0.04)	(0.04)	(0.02)
Sample N	1738	1738	1738	1738	1659	1675	1674	1738	1738

Notes: M1: climate is changing; M2: human causes of climate change; M3: climate change will harm self; M4: climate change will harm future generations; M5: support for taxes on fossil fuels; M6: subsidising renewables; M7: banning the sale of the least energy appliances; M8: discussing climate change; M9: signing a climate petition. Standard errors in parentheses.

* p < 0.05,

** p < 0.01,

*** p < 0.001.

For robustness, we rerun these bivariate models using OLS regression, controlling for a set of covariates. The results of these models remain robust and are reported in S5 Table.

Further, we examined the within-subject changes in individual’s willingness to engage in conversation about climate change in the future. Willingness to engage in conversations about climate change was measured at the start of the survey (prior to providing respondents with information about climate change and before playing the game) and again at the end of the survey. We see that playing both the placebo and climate game increases people’s self-reported intention to discuss climate change in the future. This effect is likely due to the baseline information about climate change that was provided to all respondents in the study. However, the observed change in willingness to discuss climate change is greater in the climate game condition than in the placebo (MD = 0.05, SE = 0.04; p < 0.10 statistical significance level).

### Causal mediation analyses models

**Fig 6:**
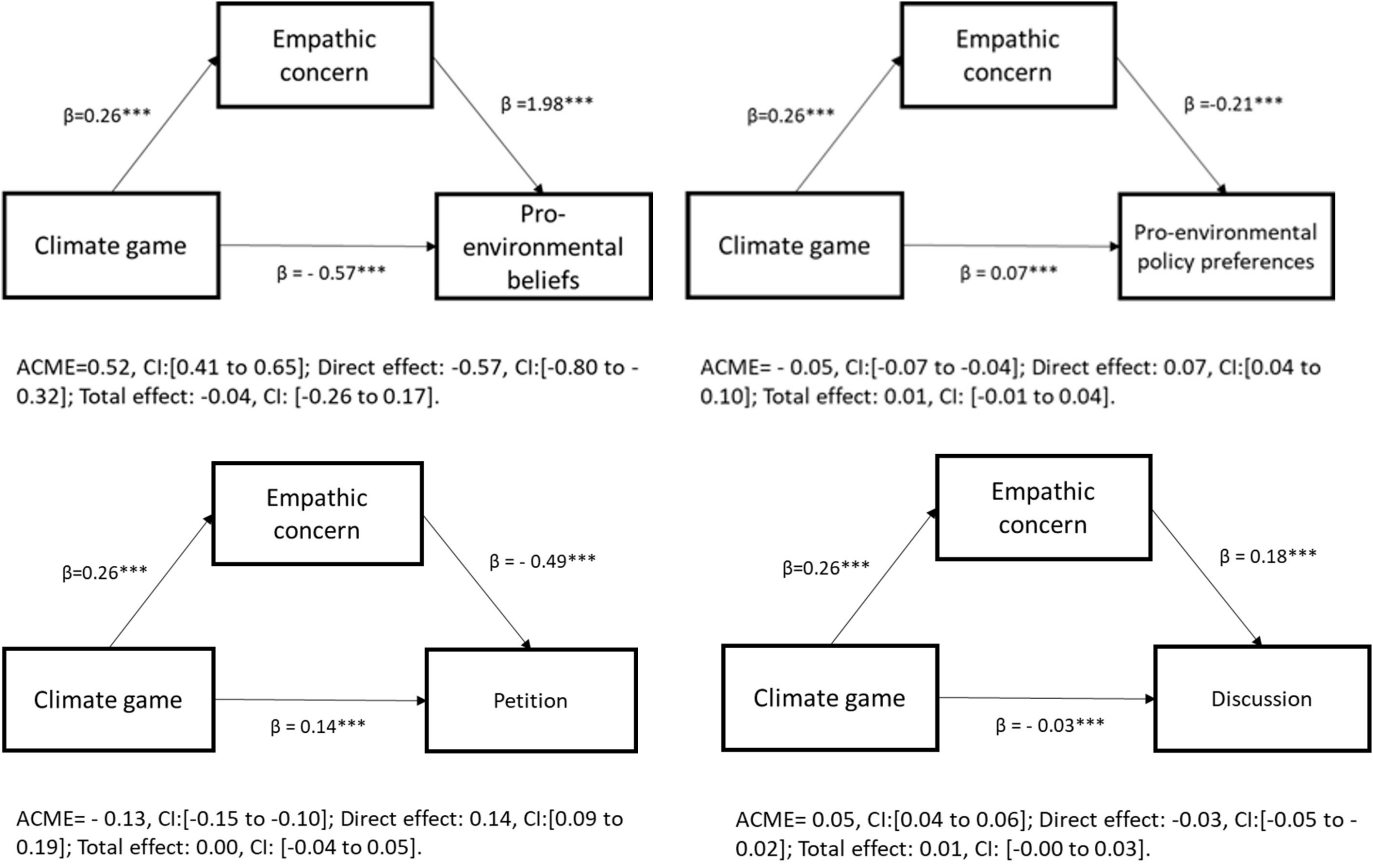
Causal mediation analyses (Study 1).

To examine the indirect effects of playing the climate change game on pro-environmental beliefs, policy support and behaviour, we conducted causal mediation analyses [34], taking the experienced “empathic concern” as a mediator and running the analyses separately for each set of dependent variables (Fig 6). For this purpose, we have created two index variables. The first sums three belief variables, whereas the second sums support for three different policy preferences. Discussion and petition were treated as separate variables. As the graph shows (top-left), the climate game has a positive and statistically significant indirect effect on pro-environmental beliefs (ACME: 0.52, CI: [0.40–0.65]; p < 0.001), that passes via the experienced empathetic concern. Disentangling the total effect into direct and indirect effects reveals that climate game exerts a significant negative direct effect on pro-environmental beliefs (β= − 0.57, CI: [−0.80 to − 0.32]). The negative direct effect and the positive indirect effect cancel each other, leading to a null total effect.

Playing the future-oriented, climate-change game has mixed effects on policy attitudes (Fig 6, top right triangle). The direct effect of playing the game on pro-climate policy attitudes is positive, albeit not substantive (direct effect: 0.07, CI: [0.04 to 0.10]). And there is no evidence that empathy indirectly mediates the effect of playing the game on policy attitudes (ACME: − 0.06, CI: [−0.07 to 0.04]).

Similar patterns are observed for the direct effect of playing the climate game on pro-climate action (Fig 6, bottom left triangle). Playing the game increases respondents’ willingness to sign a petition (direct effect: 0.14, CI: [0.09 to 0.19]), but those who feel empathic concern as a result of playing the game are less willing to sign it (ACME: − 0.13, CI: [−0.16 to − 0.10]).

Lastly, playing the climate game has the direct effect of decreasing respondents’ willingness to talk about climate change with others (−0.03, CI: [0.04 to 0.06]) (Fig 6, bottom right triangle). However, when playing the game induces empathic concern, it has the indirect effect of increasing respondents’ willingness to talk about climate change (ACME: 0.05, CI: [0.04 to 0.06]).

### Study 2 (US)

Study 2 aimed to validate the previous findings in the more polarized U.S. context and test whether the observed effect of the climate game was driven by the climate change information provided (which was constant across experimental conditions). To isolate this effect, we included a placebo game condition with no information. However, no difference was found between the placebo game with and without information in the US sample. Therefore, for simplicity and consistency with the UK study, we combined the two placebo conditions in the US sample and compared them to the treatment group.

The results show that the manipulation worked well, with respondents in the climate change game imagining their future selves more (M = 3.89; SD = 1.13; range: 1–5) than those in the placebo games (M = 3.59; SD = 1.17; two-tailed p < 0.001).

**Fig 7:**
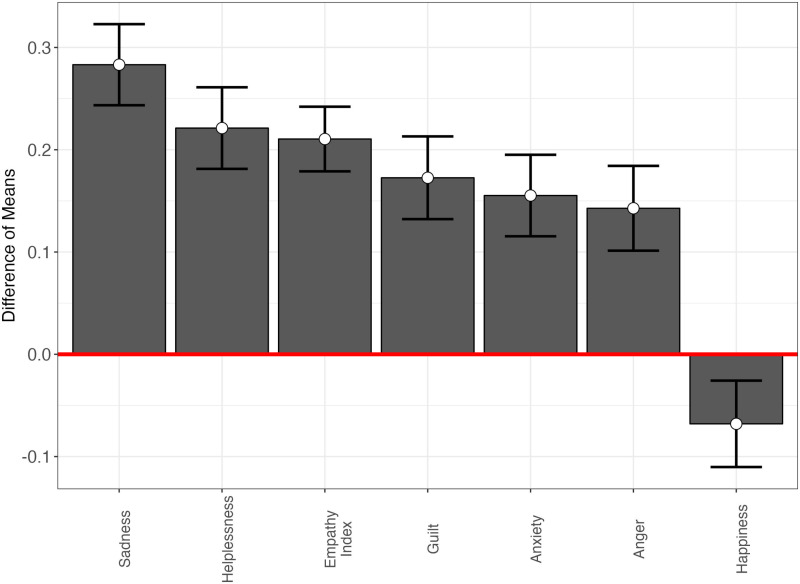
Mean differences in experienced emotions, by treatment condition (Study 2).

Furthermore, playing the climate game (as opposed to placebo games) made the American respondents much more empathetic (MD = 0.21; SE = 0.02, two-tailed p < 0.001; range: 0–1), but also more helpless, sad, guilty, anxious and angry, and less happy (see Fig 7).

Like in the UK study, the results of the OLS regression analyses that examine the H1a, b, and c show that the climate game has no main effect on subjects’ beliefs that “world’s climate is changing”, that “climate change is mainly caused by humans”, that “climate change will harm future generations”, or that “climate change will harm respondents personally”. The main effects of climate game on support for pro-environmental policies, and climate action, including people’s willingness to sign a climate petition are similarly null (see Table 4). Consistent with the findings of the UK study, playing the climate game has a positive within-subject effect on individuals’ willingness to engage in future conversations about climate change (MD = 0.20; SE = 0.04; two-tailed p < 0.001) (see also S6 Table for full models).

**Table 4:** The effects of climate game on pro-environmental beliefs, policy support and action.

	M1	M2	M3	M4	M5	M6	M7	M8	M9
Climate game	−0.13	−0.08	0.03	−0.13	0.03	−0.06	−0.07	0.05	0.02
	(0.07)	(0.05)	(0.08)	(0.07)	(0.09)	(0.09)	(0.08)	(0.08)	(0.03)
_cons	4.06^***^	3.09^***^	3.17^***^	3.8^***^	3.36^***^	2.76^***^	3.12^***^	3.11^***^	1.42^***^
	(0.04)	(0.03)	(0.05)	(0.04)	(0.05)	(0.05)	(0.05)	(0.05)	(0.02)
Sample N	1290	1290	1290	1290	1225	1228	1216	1290	1290

Notes: M1: climate is changing; M2: human causes of climate change; M3: climate change will harm self; M4: climate change will harm future generations; M5: support for taxes on fossil fuels; M6: subsidising renewables; M7: banning the sale of the least energy appliances; M8: discussing climate change; M9: signing a climate petition. Standard errors in parentheses.

* p < 0.05,

** p < 0.01,

*** p < 0.001.

### Causal mediation analyses

**Fig 8:**
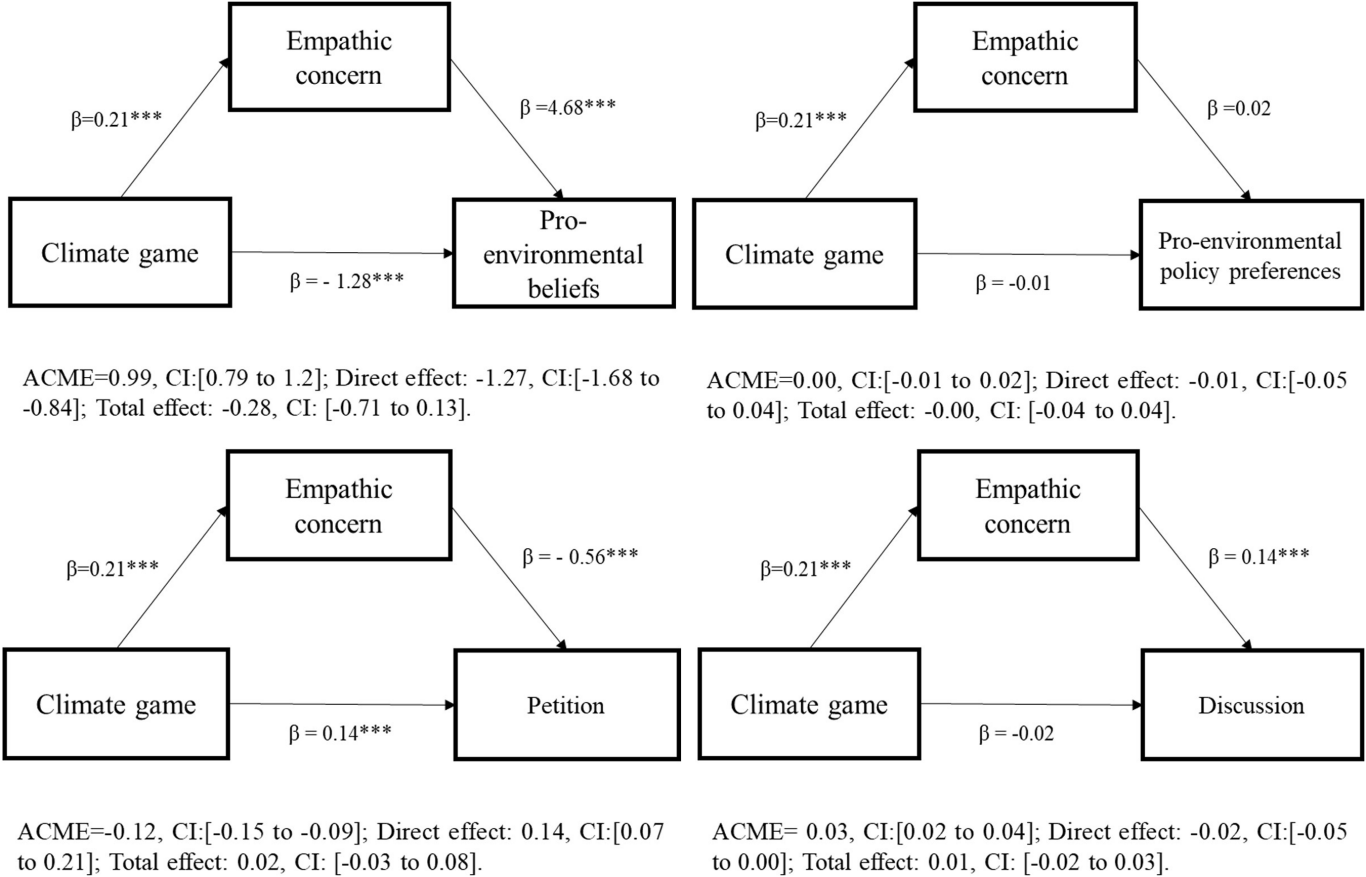
Causal mediation analyses (Study 2).

The results of the causal mediation analyses show the following patterns. Playing the climate game exerts a positive and statistically and substantively significant indirect effect on pro-environmental beliefs (ACME: 0.99, CI: [0.79 to 1.2]) and respondents’ willingness to discuss climate change with their friends and family in the future (ACME: 0.03, CI: [0.02 to 0.04]) and this relationship goes through the increased levels of empathetic concern elicited by the game. The models also show that the direct effect of climate game on pro-environmental beliefs become negative (−1.28, CI: [−1.68 to − 0.84]). There are no indirect or direct effect of climate game on Americans’ policy preferences, which differs from the UK Study results—perhaps because public policies surrounding climate change are so highly partisan in the US. However, like in the UK sample, there is a negative indirect effect of climate game on probability of Americans to sign a pro-environmental petition (ACME: − 0.12, CI: [−0.15 to 0.09]) (Fig 8).

## Discussion (Study 1 and Study 2)

Across two studies conducted in the UK and US, our project yields the following main results. First, counter to H1a, H1b and H1c, playing the climate game has no main effect on people’s beliefs about climate change, their support for environmental policies, and their self-reported behavior. However, causal mediation analyses reveal that the null effects may be due to contradictory causal processes underlying the relationship between the choose-your-own climate game and the outcomes of interest.

The following patterns are worth discussing. We find that across both samples, the climate game indirectly increases people’s pro-environmental beliefs, for instance, the idea that the climate is human-made, or that climate change will harm future generations. This indirect effect goes via experienced empathic concern. When we account for empathic concern in our models via causal mediation analyses, we also observe a negative direct effect of the climate game on pro-environmental beliefs. The negative direct effects and positive indirect effects cancel each other out, leading to null total effects. When it comes to pro-environmental policy support, the results are mixed, and mostly null. Finally, playing the choose-your-own-adventure climate game increases respondents’ willingness to sign a petition when we account for empathic concern, but those who feel empathy as a result of playing the game are less willing to sign it. Put it differently, empathy mediates the negative indirect effect of the game on signing the petition.

Finally, consistent with H2, the climate game elicits higher levels of empathic emotion in individuals. The effects are both statistically and substantively important, equaling to β=0.26 in the UK, and β=0.21 in the US sample (in a scale of 0 to 1). Expressed in standardized terms, they are equivalent to more than one standard deviation (SD) (β=1.06) among UK respondents (p < 0.001), and 0.72 SD (p < 0.001) among US respondents.

## Conclusion

This study considers whether an innovative choose-your-own-adventure story game can encourage people to take the perspective of a future self experiencing climate crises, and whether this future-oriented perspective-taking can make people more pro-environmental in their attitudes, policy support, and behaviours. The findings of this study contribute to different strands of social and behavioural science research and have implications for policymaking in the field of climate change communication, persuasion, and education.

First, playing the choose-your-own-adventure story game made individuals much more empathetic, with the effect size equaling to more than one standard deviation (SD) (β=1.06) among UK respondents (p < 0.000), and 0.72 SD among US respondents. This empathy-inducing effect of the climate game is substantively greater than the usually observed effects in social science research about perspective-taking, empathy, and opinion formation [22,23,35]. The unique value of this game lies in its ability to effectively overcome the inherent challenges of eliciting empathy for non-human subjects, such as the environment, by fostering emotional connections and encouraging perspective-taking. Scholars have recently suggested that storytelling could be a way of facilitating empathy for the nature [26]. This research adds to this scholarship, by showing that a choose-your-own-adventure game that incorporates the elements of storytelling has potential to enhance empathy in individuals. Moreover, our results show that empathy mediates the positive indirect effect of climate game on people’s pro-environmental beliefs in the UK and US. These results speak to the recent call by social scientists to place empathy at the center of policymaking on sustainability [36]. To the extent that empathy is conducive to better political judgements [24]) on different issues, including the environmental decision-making [37], fostering empathy among children and adults via choose-your-own-adventure games at schools, and beyond can have significant democratic value.

Our findings have implications for the strand of literature on climate change communication that advocates for more emotional interventions. We show that the choose-your-own-adventure game engages people’s emotions substantively, by making them much more empathetic, but also more fearful, angry and sad, among others. Moreover, the game has negative indirect effect on climate action, such as signing the climate petition. These contradictory effects of the climate game point to potential parallel causal mechanisms that underlie the relationship between perspective taking game and opinion and behaviour formation. The choose-your-own-adventure climate game exposed individuals to negative experiences – frightening and devastating climate disasters. All scenarios of the game have negative endings, irrespective of the choices players make. These scenarios may have induced higher levels of negative emotions in individuals. It is possible that hopelessness makes individuals feel that not much can be done in terms of policymaking on climate action. This corroborates the recent studies, showing that fearful representations of climate change can have backfire effects on individuals’ engagement with climate change [e.g., 18]. This finding also speaks to research that argues that in the absence of agency, empathy can lead to negative feelings of distress [36]. These results warrant a research agenda that explores more nuanced effects of emotions on attitude formation about climate change. They also have implications for climate communication policymaking. While exposing individuals to climate disasters may be more realistic, the emotional and behavioral consequences of such exposure could be negative. Therefore, care is needed in designing such interventions.

We also contribute to larger political science scholarship that investigates the role of emotions in political cognition. A recent review suggests capturing emotions experienced simultaneously in response to socio-political stimuli [38]. By measuring a range of emotions individuals experienced having played the climate game, we demonstrate that exposure to a treatment can elicit diverse emotional reactions in subjects. Capturing and controlling for these emotions is crucial to our understanding of how individuals engage with climate change and environmental information.

Moreover, our study has implications for political communication on climate change. In the UK sample only, we find that playing the climate game increases respondents’ reported intention to talk about climate change in the future. Discussing climate change is one way of making it a salient issue for people’s lives and can motivate personal and collective action [39,40]. This study shows that playing this climate game can enhance people’s intention to talk about climate change.

### Limitations and future research

One limitation of our current study is that all scenarios in our choose-your-own-adventure climate game end negatively. As such, we only show the effects of future-oriented thinking and empathic concern on environmental beliefs, attitudes, and action in a series of negative scenarios. While this might be more “realistic” in the sense that climate change does and will produce a range of negative outcomes that no individual person has the power to stop by their own volition, future studies might consider how allowing participants to play the role of a hero who has the power to change the course of history in a fictional game can yield more positive results. Empowering participants to play a character who can produce positive scenarios in the face of climate disasters may have different effects on climate-related beliefs, attitudes, and actions.

Another limitation of our present work is that we do not capture the causal mechanism by which the direct effect of playing the climate game reduces support for pro-climate policies and suppresses actions to address climate change. While we speculate that demobilization is caused by hopelessness, future studies should test this link with more sophisticated designs.

### Concluding thoughts

Despite the shortcomings, our results show that absent the indirect, mediating effect of empathy, potential negative experiences of having been exposed to climate crises may leave individuals feeling hopeless and overwhelmed. Like in our game, climate change is causing a series of disasters resulting in negative outcomes for millions of people. These negative experiences directly increase the belief that climate change is happening. However, in the absence of empathic concern for future generations, these overwhelming negative experiences may reduce support for pro-climate policies. To mitigate and cancel out this demobilization, policymakers should harness the power of future-oriented thinking to indirectly boost pro-climate attitudes and action by increasing empathic concern for future generations. While the net effect of inducing empathic concern from playing a future-oriented game was not enough to fully overcome the direct consequences of suffering a series of climate disasters in our game, in some cases it is able to cancel out the demobilizing effects of the overwhelmingly negative consequences of climate change.

Lastly, with this study we provide a blueprint for eliciting empathy in individuals towards the environment using a first-person, choose-you-own-adventure game. This is an online, low-cost, and scalable intervention that is entertaining and easier to introduce in real-world. The game holds significant practical value, especially in the field of environmental education and activism and could also influence the development of other social marketing strategies and initiatives.

## Supporting Information

S1 Fig Example Scenarios for the Climate Game – UK (word)

S1 Table Descriptive Sample Statistics (UK) (word)

S2 Table Descriptive Sample Statistics (US) (word)

S3 Table Variable Means and Standard Deviations (UK) (word)

S4 Table Variable Means and Standard Deviations (US) (word)

S5 Table Full Models (UK) (word)

S6 Table Full Models (US) (word)

S1 File List of deviations from the pre-registration (Word)
