# Supplementary material for: Promoting Pro-environmental Beliefs and Behaviour: Choose-Your-Own Story Futuristic Climate Game
Source: PLoS One. 2025 Mar 31;20(3):e0317773. doi: 10.1371/journal.pone.0317773 (PMC11957362; doi:10.1371/journal.pone.0317773)
Supplement: S1 Fig — (word) [file pone.0317773.s001.docx]

# S1 Fig. Example Scenarios for the Climate Game – UK

(with screenshots of Qualtrics design of the game)


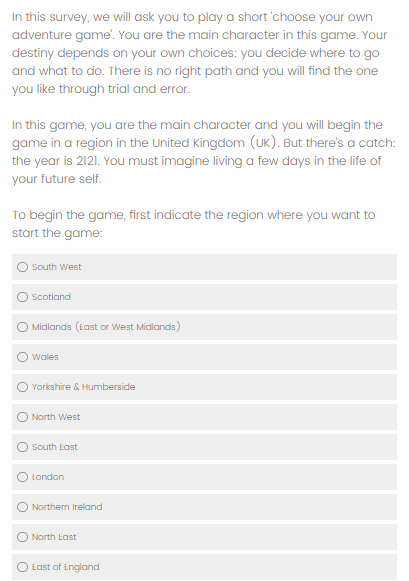


Depending on the chosen region, individuals will be guided to one of five modules (Fire, Flood, Erosion, Drought or Famine). Below we give examples from floods module.


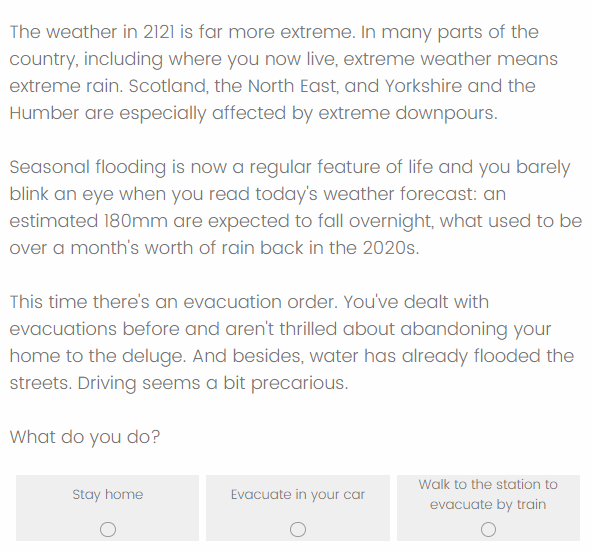


If the respondent chooses, “stay at home”, then they are presented with the following outcome and choice.


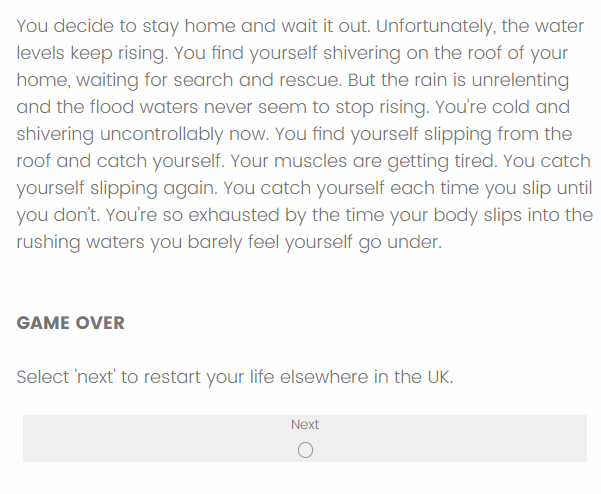


If the respondent clicks on “evacuate in your car”, they are presented with the following situation and choice.


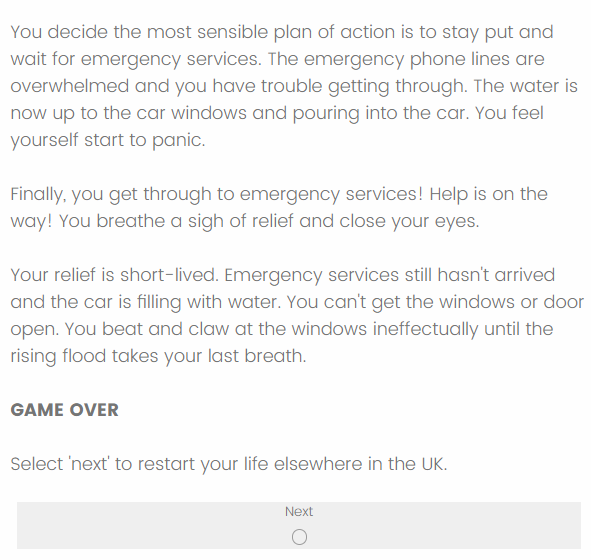


The game is played until all modules have been read and choices have been made. The end if “game over”.
