# Supplementary material for: Promoting Pro-environmental Beliefs and Behaviour: Choose-Your-Own Story Futuristic Climate Game
Source: PLoS One. 2025 Mar 31;20(3):e0317773. doi: 10.1371/journal.pone.0317773 (PMC11957362; doi:10.1371/journal.pone.0317773)
Supplement: S1 Table — (word) [file pone.0317773.s002.docx]

# S1 Table. Descriptive Sample Statistics (UK)

| **Table 1.** | | |
| --- | --- | --- |
|  | Count | % |
| **gender** | | |
| Man | 808 | 46.5 |
| Woman | 925 | 53.3 |
| Trans, non-binary or another gender identity | 4 | 0.2 |
| Total | 1,737 | 100 |
| **education** | | |
| Did not finish secondary school | 31 | 1.8 |
| Finished secondary school | 672 | 38.7 |
| Finished 3rd level | 714 | 41.1 |
| Masters/PhD | 320 | 18.4 |
| Total | 1,737 | 100 |
| **party** | | |
| Labour | 467 | 26.9 |
| Conservative | 655 | 37.7 |
| Liberal Democrat | 169 | 9.7 |
| UKIP | 32 | 1.8 |
| SNP | 47 | 2.7 |
| Green Party of England and Wales | 84 | 4.8 |
| Scottish Green Party | 7 | 0.4 |
| Green Party in Northern Ireland | 9 | 0.5 |
| Other | 53 | 3 |
| Don't know/None | 215 | 12.4 |
| Total | 1,738 | 100 |
| **region** | | |
| East Anglia | 151 | 8.7 |
| East Midlands | 123 | 7.1 |
| London | 221 | 12.7 |
| North East | 82 | 4.7 |
| North West | 189 | 10.9 |
| Northern Ireland | 33 | 1.9 |
| Scotland | 140 | 8.1 |
| South East | 250 | 14.4 |
| South West | 155 | 8.9 |
| Wales | 91 | 5.2 |
| West Midlands | 155 | 8.9 |
| Yorkshire & Humberside | 147 | 8.5 |
| Other | 1 | 0.1 |
| Total | 1,738 | 100 |
