# Supplementary material for: Promoting Pro-environmental Beliefs and Behaviour: Choose-Your-Own Story Futuristic Climate Game
Source: PLoS One. 2025 Mar 31;20(3):e0317773. doi: 10.1371/journal.pone.0317773 (PMC11957362; doi:10.1371/journal.pone.0317773)
Supplement: S2 Table — (word) [file pone.0317773.s003.docx]

# S2 Table. Descriptive Sample Statistics (US)

| **Table 1.** | | |
| --- | --- | --- |
|  | Count | Sample % |
| **gender** | | |
| Man | 600 | 46.5 |
| Woman | 679 | 52.6 |
| Trans, non-binary or another gender identity | 11 | 0.9 |
| Total | 1,290 | 100 |
| **education** | | |
| less than high school | 107 | 8.3 |
| high school or equivalent | 558 | 43.3 |
| some college or uni, no degree | 130 | 10.1 |
| associate degree in college - occupational/vocational | 60 | 4.7 |
| associate degree - academic | 39 | 3 |
| bachelor | 244 | 18.9 |
| postgraduate degree | 151 | 11.7 |
| Total | 1,289 | 100 |
| **party** | | |
| democrat | 574 | 46.2 |
| republican | 355 | 28.6 |
| independent | 284 | 22.9 |
| other | 29 | 2.3 |
| Total | 1,242 | 100 |
| **State^+^** | | |
| Alabama | 18 | 1.4 |
| Alaska | 3 | 0.2 |
| Arizona | 34 | 2.6 |
| Arkansas | 10 | 0.8 |
| California | 103 | 8 |
| Colorado | 12 | 0.9 |
| Connecticut | 10 | 0.8 |
| Delaware | 2 | 0.2 |
| District of Columbia | 2 | 0.2 |
| Florida | 107 | 8.3 |
| Georgia | 42 | 3.3 |
| Hawaii | 10 | 0.8 |
| Idaho | 4 | 0.3 |
| Illinois | 55 | 4.3 |
| Indiana | 21 | 1.6 |
| Iowa | 10 | 0.8 |
| Kansas | 13 | 1 |
| Kentucky | 24 | 1.9 |
| Louisiana | 16 | 1.2 |
| Maine | 2 | 0.2 |
| Maryland | 9 | 0.7 |
| Massachusetts | 29 | 2.2 |
| Michigan | 47 | 3.6 |
| Minnesota | 11 | 0.9 |
| Mississippi | 11 | 0.9 |
| Missouri | 35 | 2.7 |
| Montana | 4 | 0.3 |
| Nebraska | 14 | 1.1 |
| Nevada | 32 | 2.5 |
| New Hampshire | 6 | 0.5 |
| New Jersey | 48 | 3.7 |
| New Mexico | 3 | 0.2 |
| New York | 113 | 8.8 |
| North Carolina | 42 | 3.3 |
| North Dakota | 1 | 0.1 |
| Ohio | 65 | 5 |
| Oklahoma | 14 | 1.1 |
| Oregon | 15 | 1.2 |
| Pennsylvania | 68 | 5.3 |
| Rhode Island | 2 | 0.2 |
| South Carolina | 15 | 1.2 |
| South Dakota | 1 | 0.1 |
| Tennessee | 24 | 1.9 |
| Texas | 77 | 6 |
| Utah | 7 | 0.5 |
| Vermont | 1 | 0.1 |
| Virginia | 27 | 2.1 |
| Washington | 16 | 1.2 |
| West Virginia | 7 | 0.5 |
| Wisconsin | 44 | 3.4 |
| Wyoming | 1 | 0.1 |
| I do not reside in the United States | 2 | 0.2 |
| Total | 1,289 | 100 |
