# Supplementary material for: Promoting Pro-environmental Beliefs and Behaviour: Choose-Your-Own Story Futuristic Climate Game
Source: PLoS One. 2025 Mar 31;20(3):e0317773. doi: 10.1371/journal.pone.0317773 (PMC11957362; doi:10.1371/journal.pone.0317773)
Supplement: S3 Table — (word) [file pone.0317773.s004.docx]

# S3 Table. Variable Means and Standard Deviations (UK)

|  | | |
| --- | --- | --- |
|  | sample mean | sample SD |
| climate is changing | 4.30 | 0.90 |
| human causes of climate change | 3.31 | 0.72 |
| harm for future gen's | 3.95 | 1.05 |
| Self-harm | 2.80 | 1.09 |
| policypref_1 | 2.52 | 1.26 |
| policypref_2 | 2.09 | 1.13 |
| policypref_3 | 2.25 | 1.15 |
| signing a petition | 1.48 | 0.50 |
| intention to discuss climate change (post-treatment) | 2.89 | 1.19 |
| Observations | 1738 |  |
