# Supplementary material for: Promoting Pro-environmental Beliefs and Behaviour: Choose-Your-Own Story Futuristic Climate Game
Source: PLoS One. 2025 Mar 31;20(3):e0317773. doi: 10.1371/journal.pone.0317773 (PMC11957362; doi:10.1371/journal.pone.0317773)
Supplement: S4 Table — (word) [file pone.0317773.s005.docx]

# S4 Table. Variable Means and Standard Deviations (US)

|  | | |
| --- | --- | --- |
|  | sample mean | sample SD |
| climate is changing | 4.02 | 1.18 |
| human causes of climate change | 3.06 | 0.87 |
| harm for future gen's | 3.77 | 1.26 |
| Self-harm | 3.18 | 1.32 |
| policypref_1 | 3.37 | 1.50 |
| policypref_2 | 2.74 | 1.47 |
| policypref_3 | 3.09 | 1.40 |
| signing a petition | 1.43 | 0.50 |
| intention to discuss climate change (pre-treatment) | 2.90 | 1.40 |
| intention to discuss climate change (post-treatment) | 3.12 | 1.38 |
| Sample N | 1290 |  |
|  |  |  |
