# Supplementary material for: Promoting Pro-environmental Beliefs and Behaviour: Choose-Your-Own Story Futuristic Climate Game
Source: PLoS One. 2025 Mar 31;20(3):e0317773. doi: 10.1371/journal.pone.0317773 (PMC11957362; doi:10.1371/journal.pone.0317773)
Supplement: S5 Table — (word) [file pone.0317773.s006.docx]

# S5 Table. Full Models (UK)

|  | | | | | | | | | |
| --- | --- | --- | --- | --- | --- | --- | --- | --- | --- |
|  | M1 | M2 | M3 | M4 | M5 | M6 | M7 | M8 | M9 |
| climate game | -0.0260 | -0.0125 | 0.0657 | -0.0237 | 0.0452 | 0.0319 | 0.0653 | 0.0490 | 0.00253 |
|  | (0.0414) | (0.0332) | (0.0481) | (0.0490) | (0.0602) | (0.0545) | (0.0558) | (0.0543) | (0.0236) |
| left-right | -0.0761*** | -0.0690*** | 0.00235 | -0.0739*** | 0.0912*** | 0.0748*** | 0.0510*** | 0.0113 | -0.0121 |
|  | (0.0111) | (0.00888) | (0.0129) | (0.0131) | (0.0159) | (0.0145) | (0.0149) | (0.0145) | (0.00631) |
| party: conservative (ref: labour) | -0.0791 | 0.0180 | -0.367*** | -0.289*** | 0.160 | 0.0831 | 0.156 | -0.345*** | 0.199*** |
|  | (0.0621) | (0.0498) | (0.0720) | (0.0733) | (0.0901) | (0.0814) | (0.0831) | (0.0813) | (0.0354) |
| party: liberal democrat | -0.0490 | 0.113 | -0.170 | 0.00429 | -0.0582 | -0.0580 | 0.0405 | -0.0831 | 0.103* |
|  | (0.0782) | (0.0627) | (0.0907) | (0.0924) | (0.114) | (0.103) | (0.105) | (0.102) | (0.0445) |
| party: ukip | -0.339* | -0.227 | -0.406* | -0.476* | 0.384 | 0.354 | 0.353 | -0.434* | 0.107 |
|  | (0.160) | (0.129) | (0.186) | (0.189) | (0.235) | (0.211) | (0.215) | (0.210) | (0.0914) |
| party: snp | -0.0279 | 0.178 | -0.365* | -0.117 | 0.189 | -0.00970 | -0.00650 | -0.111 | -0.0958 |
|  | (0.131) | (0.105) | (0.152) | (0.155) | (0.190) | (0.173) | (0.175) | (0.171) | (0.0746) |
| party: green party | 0.231* | 0.220** | 0.0637 | 0.212 | -0.412** | -0.337* | -0.404** | 0.207 | -0.136* |
|  | (0.102) | (0.0819) | (0.119) | (0.121) | (0.147) | (0.135) | (0.137) | (0.134) | (0.0582) |
| party: scottish green | 0.300 | 0.0127 | 1.120** | 0.488 | -0.355 | 0.0375 | -0.529 | 0.980* | -0.174 |
|  | (0.325) | (0.261) | (0.377) | (0.384) | (0.462) | (0.420) | (0.430) | (0.426) | (0.185) |
| party: green party of northern ireland | 0.297 | 0.0178 | -0.245 | -0.376 | 0.532 | 0.178 | 0.225 | -0.0318 | -0.256 |
|  | (0.287) | (0.230) | (0.333) | (0.339) | (0.408) | (0.372) | (0.402) | (0.376) | (0.164) |
| party: other | -0.193 | -0.0203 | -0.413** | -0.224 | 0.120 | 0.369* | 0.286 | -0.360* | 0.217** |
|  | (0.127) | (0.102) | (0.148) | (0.150) | (0.183) | (0.168) | (0.172) | (0.166) | (0.0725) |
| party: don't know | -0.252*** | -0.0808 | -0.419*** | -0.386*** | 0.370*** | 0.332*** | 0.195 | -0.675*** | 0.236*** |
|  | (0.0728) | (0.0584) | (0.0845) | (0.0860) | (0.109) | (0.0986) | (0.101) | (0.0953) | (0.0415) |
| age | 0.00600*** | 0.00219 | -0.0214*** | -0.00136 | 0.00172 | -0.00590** | -0.00281 | -0.0128*** | 0.00125 |
|  | (0.00140) | (0.00113) | (0.00163) | (0.00166) | (0.00205) | (0.00185) | (0.00190) | (0.00184) | (0.000800) |
| gender: woman | 0.131** | 0.0573 | 0.130** | 0.133** | -0.0783 | -0.0358 | -0.0956 | -0.0142 | -0.00839 |
|  | (0.0423) | (0.0339) | (0.0491) | (0.0499) | (0.0614) | (0.0557) | (0.0570) | (0.0554) | (0.0241) |
| gender: other | 0.483 | 0.173 | -0.280 | -0.299 | -0.0364 | -0.577 | 0.697 | 0.334 | 0.0452 |
|  | (0.430) | (0.345) | (0.499) | (0.508) | (0.611) | (0.556) | (0.568) | (0.563) | (0.245) |
| edu: secondary edu | 0.0137 | 0.342** | -0.376* | -0.284 | 0.379 | 0.0756 | -0.0605 | -0.249 | 0.0342 |
|  | (0.161) | (0.129) | (0.186) | (0.190) | (0.240) | (0.227) | (0.224) | (0.210) | (0.0915) |
| edu: 3rd level | 0.122 | 0.444*** | -0.361 | -0.214 | 0.286 | -0.0162 | -0.220 | -0.0302 | 0.0513 |
|  | (0.160) | (0.129) | (0.186) | (0.190) | (0.240) | (0.227) | (0.224) | (0.210) | (0.0914) |
| edu: postgraduate | 0.151 | 0.550*** | -0.111 | -0.0868 | 0.0257 | -0.0899 | -0.319 | 0.312 | -0.0448 |
|  | (0.165) | (0.132) | (0.192) | (0.195) | (0.247) | (0.232) | (0.230) | (0.216) | (0.0941) |
| race: another white background | -0.0341 | -0.0277 | -0.0161 | -0.112 | 0.0596 | 0.00437 | 0.201 | 0.0515 | -0.0674 |
|  | (0.0908) | (0.0728) | (0.105) | (0.107) | (0.130) | (0.118) | (0.121) | (0.119) | (0.0518) |
| race: white and black caribbean | -0.176 | -0.00244 | -0.341 | 0.0211 | 0.291 | -0.0102 | 0.0128 | -0.367 | 0.135 |
|  | (0.166) | (0.133) | (0.193) | (0.196) | (0.245) | (0.223) | (0.228) | (0.217) | (0.0946) |
| race: white and black african | -0.353 | -0.277 | -0.0482 | -0.232 | -0.466 | -0.230 | 0.0828 | -0.201 | 0.148 |
|  | (0.197) | (0.158) | (0.229) | (0.233) | (0.296) | (0.269) | (0.276) | (0.258) | (0.112) |
| race: white and asian | 0.0460 | 0.134 | 0.104 | 0.130 | -0.0676 | -0.0311 | 0.0683 | 0.100 | -0.120 |
|  | (0.122) | (0.0977) | (0.141) | (0.144) | (0.173) | (0.159) | (0.164) | (0.160) | (0.0694) |
| race: another mixed | 0.0979 | -0.252 | 0.0524 | 0.312 | 0.443 | -0.0399 | 0.410 | -0.0510 | 0.205 |
|  | (0.248) | (0.199) | (0.288) | (0.293) | (0.368) | (0.335) | (0.343) | (0.325) | (0.141) |
| race: indian | -0.191 | -0.167 | 0.725*** | 0.144 | 0.0308 | 0.359 | -0.0750 | 0.314 | -0.225** |
|  | (0.149) | (0.119) | (0.173) | (0.176) | (0.218) | (0.198) | (0.203) | (0.195) | (0.0849) |
| race: pakistani | -0.597** | -0.181 | 0.413 | -0.154 | 0.103 | 0.239 | -0.0507 | 0.529* | -0.104 |
|  | (0.206) | (0.165) | (0.239) | (0.243) | (0.293) | (0.274) | (0.280) | (0.269) | (0.117) |
| race: bangladeshi | -0.312 | 0.210 | 0.232 | -0.178 | 0.379 | 0.610 | 0.301 | -0.326 | 0.0568 |
|  | (0.351) | (0.281) | (0.407) | (0.414) | (0.498) | (0.454) | (0.464) | (0.459) | (0.200) |
| race: another asian background | -0.0759 | 0.0478 | 0.243 | 0.0620 | 0.354 | -0.382 | -0.0771 | -0.0799 | -0.0772 |
|  | (0.287) | (0.230) | (0.332) | (0.339) | (0.407) | (0.371) | (0.379) | (0.375) | (0.163) |
| race:black caribbean | -0.658** | -0.401* | -0.481 | -0.425 | 0.524 | 0.697* | 0.438 | -0.148 | 0.00811 |
|  | (0.241) | (0.193) | (0.279) | (0.284) | (0.356) | (0.324) | (0.346) | (0.315) | (0.137) |
| race: black african | -0.239 | -0.496*** | -0.278 | -0.226 | 0.880*** | 0.580* | 0.826*** | -0.102 | -0.168 |
|  | (0.178) | (0.143) | (0.207) | (0.211) | (0.254) | (0.231) | (0.236) | (0.233) | (0.102) |
| race: another black | -1.642*** | -1.659*** | -0.960 | -0.907 | 2.104** | 0.905 | 0.884 | -1.513* | 0.332 |
|  | (0.495) | (0.396) | (0.574) | (0.584) | (0.703) | (0.639) | (0.654) | (0.648) | (0.282) |
| race: chinese | -0.0340 | -0.121 | 0.171 | 0.00489 | 0.348 | 0.672* | 0.334 | -0.621* | -0.148 |
|  | (0.224) | (0.179) | (0.260) | (0.264) | (0.330) | (0.300) | (0.307) | (0.293) | (0.128) |
| race: another racial/ethnic group# | -0.383 | 0.257 | -0.0671 | 0.132 | -0.230 | -0.253 | -0.313 | -0.393 | -0.0546 |
|  | (0.303) | (0.243) | (0.352) | (0.358) | (0.460) | (0.419) | (0.464) | (0.397) | (0.173) |
| race: don't now/prefer not to | -0.286 | -0.183 | -0.208 | -0.355 | 0.503 | 0.553 | 0.237 | -0.500 | 0.202 |
|  | (0.240) | (0.192) | (0.278) | (0.283) | (0.369) | (0.336) | (0.344) | (0.314) | (0.137) |
| _cons | 4.372*** | 3.139*** | 4.290*** | 4.746*** | 1.558*** | 1.887*** | 2.187*** | 3.740*** | 1.360*** |
|  | (0.186) | (0.149) | (0.215) | (0.219) | (0.277) | (0.258) | (0.258) | (0.243) | (0.106) |
| N | 1721 | 1721 | 1721 | 1721 | 1643 | 1659 | 1658 | 1721 | 1721 |
| R-sq | 0.109 | 0.117 | 0.201 | 0.098 | 0.091 | 0.077 | 0.059 | 0.141 | 0.078 |
| Note: M1: climate is changing; M2: human causes of climate change; M3: climate change will harm self; M4: climate change will harm future generations; M5: support for taxes on fossil fuels; M6: subsidising renewables; M7: banning the sale of the least energy appliances; M8: discussing climate change; M9: signing a climate petition. Standard errors in parentheses. * p<0.05, ** p<0.01, *** p<0.001. | | | | | | | | | |
