# Supplementary material for: Promoting Pro-environmental Beliefs and Behaviour: Choose-Your-Own Story Futuristic Climate Game
Source: PLoS One. 2025 Mar 31;20(3):e0317773. doi: 10.1371/journal.pone.0317773 (PMC11957362; doi:10.1371/journal.pone.0317773)
Supplement: S6 Table — (word) [file pone.0317773.s007.docx]

# S6 Table. Full Models (US)

|  | | | | | | | | | |
| --- | --- | --- | --- | --- | --- | --- | --- | --- | --- |
|  | M1 | M2 | M3 | M4 | M5 | M6 | M7 | M8 | M9 |
| climate game | -0.06 | -0.0414 | 0.0216 | -0.0929 | -0.0245 | -0.120 | -0.0970 | 0.0433 | 0.0291 |
|  | (0.07) | (0.0495) | (0.0680) | (0.0696) | (0.0867) | (0.0835) | (0.0818) | (0.0687) | (0.0271) |
| left-right | -0.08*** | -0.0626*** | 0.0338** | -0.0635*** | 0.156*** | 0.184*** | 0.130*** | 0.0559*** | -0.01 |
|  | (0.01) | (0.00869) | (0.0120) | (0.0122) | (0.0152) | (0.0146) | (0.0144) | (0.0121) | (0.01) |
| party: republican (ref: democrat) | -0.61*** | -0.352*** | -1.083*** | -0.934*** | 0.198 | 0.0513 | 0.219* | -1.034*** | 0.26*** |
|  | (0.08) | (0.0617) | (0.0848) | (0.0867) | (0.107) | (0.103) | (0.101) | (0.0856) | (0.03) |
| party: independent | -0.45*** | -0.315*** | -0.731*** | -0.602*** | 0.123 | -0.148 | 0.0762 | -0.775*** | 0.228*** |
|  | (0.08) | (0.0612) | (0.0842) | (0.0861) | (0.109) | (0.104) | (0.102) | (0.0850) | (0.0336) |
| party: other | -0.45* | -0.327* | -0.697** | -0.699** | 0.196 | 0.238 | 0.578* | -0.716** | 0.286** |
|  | (0.22) | (0.163) | (0.224) | (0.229) | (0.288) | (0.282) | (0.277) | (0.226) | (0.0893) |
| age | -0.00 | -0.00243 | -0.0227*** | -0.00828*** | 0.00688** | 0.00565* | 0.00417 | -0.0227*** | 0.00663*** |
|  | (0.00) | (0.00149) | (0.00205) | (0.00210) | (0.00261) | (0.00250) | (0.00246) | (0.00207) | (0.00) |
| gender: woman (ref: man) | 0.03 | -0.118* | -0.0769 | -0.0405 | -0.247** | -0.154 | -0.230** | -0.164* | 0.12*** |
|  | (0.07) | (0.0490) | (0.0673) | (0.0688) | (0.0859) | (0.0820) | (0.0807) | (0.0680) | (0.03) |
| gender: other | 0.36 | -0.0290 | -0.131 | 0.100 | -0.404 | -0.373 | -0.364 | -0.271 | 0.06 |
|  | (0.36) | (0.269) | (0.370) | (0.378) | (0.461) | (0.443) | (0.457) | (0.373) | (0.15) |
| edu: high school | 0.10 | 0.199* | -0.243 | 0.0670 | 0.0838 | -0.0493 | -0.0742 | -0.0606 | 0.06 |
|  | (0.12) | (0.0910) | (0.125) | (0.128) | (0.163) | (0.153) | (0.151) | (0.126) | (0.05) |
| edu: some college, no degree | 0.23 | 0.248* | -0.0522 | 0.283 | 0.135 | -0.222 | -0.0448 | 0.301 | 0.03 |
|  | (0.15) | (0.112) | (0.154) | (0.158) | (0.198) | (0.188) | (0.185) | (0.156) | (0.06) |
| edu: occupational | -0.08 | 0.126 | -0.269 | -0.291 | 0.288 | 0.0150 | 0.0747 | -0.000742 | 0.05 |
|  | (0.19) | (0.137) | (0.189) | (0.193) | (0.243) | (0.232) | (0.225) | (0.191) | (0.0754) |
| edu: associate deg | 0.20 | 0.205 | 0.00903 | -0.0442 | -0.766** | -0.821** | -0.653* | 0.473* | 0.0496 |
|  | (0.21) | (0.156) | (0.215) | (0.220) | (0.277) | (0.259) | (0.258) | (0.217) | (0.0857) |
| edu: bachelor | 0.46*** | 0.496*** | -0.00509 | 0.300* | 0.00546 | 0.0441 | 0.0470 | 0.483*** | -0.0223 |
|  | (0.13) | (0.100) | (0.138) | (0.141) | (0.178) | (0.167) | (0.165) | (0.139) | (0.0549) |
| edu: postgraduate | 0.22 | 0.275* | 0.167 | 0.390* | -0.750*** | -0.612*** | -0.780*** | 0.640*** | -0.0648 |
|  | (0.15) | (0.108) | (0.149) | (0.152) | (0.191) | (0.181) | (0.177) | (0.150) | (0.0592) |
| race: black/afro-american | -0.18 | -0.174* | -0.0757 | -0.257* | 0.108 | 0.201 | 0.0102 | -0.0264 | -0.0660 |
|  | (0.11) | (0.0783) | (0.108) | (0.110) | (0.139) | (0.132) | (0.132) | (0.109) | (0.0429) |
| race: hispanic/latino | -0.02 | -0.0519 | -0.309 | -0.333 | -0.154 | 0.168 | -0.120 | -0.263 | 0.108 |
|  | (0.12) | (0.123) | (0.169) | (0.173) | (0.219) | (0.206) | (0.209) | (0.171) | (0.0675) |
| race: native american | 0.07 | 0.374 | 0.145 | 0.485 | -0.117 | -0.881 | -0.372 | 0.578 | -0.179 |
|  | (0.37) | (0.277) | (0.381) | (0.389) | (0.502) | (0.516) | (0.473) | (0.384) | (0.152) |
| race: middle easter | -0.11 | -0.247 | 0.399 | 0.274 | -0.372 | -0.832 | -1.362* | 0.762 | -0.114 |
|  | (0.497) | (0.368) | (0.506) | (0.518) | (0.630) | (0.605) | (0.593) | (0.511) | (0.202) |
| race: asian | 0.0802 | 0.179 | -0.0933 | -0.219 | 0.0579 | -0.254 | 0.0227 | -0.188 | 0.127 |
|  | (0.167) | (0.124) | (0.170) | (0.174) | (0.220) | (0.205) | (0.203) | (0.172) | (0.0677) |
| race: mixed | -0.217 | -0.0135 | -0.304 | -0.404 | 0.125 | 0.0566 | -0.348 | -0.122 | 0.151 |
|  | (0.210) | (0.155) | (0.214) | (0.218) | (0.285) | (0.278) | (0.263) | (0.216) | (0.0852) |
| race: native hawaiian | 0.249 | -0.200 | 1.449* | 0.464 | -1.379 | -1.174 | -2.055** | 1.148* | -0.117 |
|  | (0.556) | (0.412) | (0.566) | (0.579) | (0.704) | (0.677) | (0.662) | (0.572) | (0.226) |
| race: another | -0.861 | -0.797 | -0.598 | -1.181 | 0.151 | -0.811 | -0.289 | -0.176 | 0.122 |
|  | (0.640) | (0.474) | (0.652) | (0.666) | (0.810) | (0.779) | (0.762) | (0.658) | (0.260) |
| race: don't know | -1.163* | -0.224 | -0.338 | -0.852 | 0.792 | 0.234 | 0.734 | 0.601 | 0.00608 |
|  | (0.498) | (0.369) | (0.508) | (0.519) | (0.631) | (0.607) | (0.594) | (0.513) | (0.202) |
| _cons | 4.722*** | 3.606*** | 4.692*** | 4.954*** | 2.123*** | 1.572*** | 2.283*** | 4.230*** | 0.924*** |
|  | (0.185) | (0.137) | (0.189) | (0.193) | (0.244) | (0.230) | (0.228) | (0.191) | (0.0753) |
| N | 1223 | 1223 | 1223 | 1223 | 1164 | 1166 | 1157 | 1223 | 1223 |
| R-sq | 0.143 | 0.141 | 0.297 | 0.199 | 0.161 | 0.191 | 0.150 | 0.345 | 0.195 |
| Note: M1: climate is changing; M2: human causes of climate change; M3: climate change will harm self; M4: climate change will harm future generations; M5: support for taxes on fossil fuels; M6: subsidising renewables; M7: banning the sale of the least energy appliances; M8: discussing climate change; M9: signing a climate petition. Standard errors in parentheses. * p<0.05, ** p<0.01, *** p<0.001. | | | | | | | | | |

# List of Deviations from the Pre-registration

The study was initially preregistered (AsPredicted #54003) on 10/12/2020, and then further amended twice (#58539, 17/02/2021, and #58592, 17/02/2021). As such, the research questions and hypotheses guiding this project are in three different documents. All these amendments happened prior to data collection. Below we show the list of main deviations from these preregistration plans.

- In the original preregistration plan, we also intended to test another hypothesis, which stated that “Those respondents who hold more ambivalent (i.e. non-extreme, middle-of-the road) attitudes on environment will be more strongly affected by perspective taking intervention.” Due to space restrictions and in order not to convolute the paper, we decided to leave this hypothesis for another, shorter paper.
- There was also an additional research question, which intended to study the durability of the (potential) effects. Due to scarce resources, we decided against fielding a follow up survey.
- The original plan said that we would control for dispositional empathy, and whether the person has a child or not, in some of our models. We opted for not including those questions due to space restrictions in the survey.
- In the section 8 of the pre-registration plan, we list other exploratory analyses we were intending to do. We conduct some relevant ones either within the MS or the online appendix. We are not, however, reporting all of them in this paper, mostly because we ended up not including some of those questions (e.g., see the previous point) in the latest version of the survey.
- In one of the amendments, we hypothesize about mediating role of another emotion – anxiety – captured via the emotion word of “fear”. We expect that distressing situations depicted in the climate game may simultaneously heighten people’s feelings of anxiety and hopelessness, potentially producing conflicting effects on our outcome variables. We leave this hypothesis for another paper.
- In the original pre-registration plan, we state that we would explore the mediating effect of empathic concern and would conduct causal mediation analyses. In the body of this manuscript, we present this as exploratory hypotheses, by saying this: “Third, we explore whether the choose-your-own-adventure game would specifically increase individuals' empathetic concern (H3a) and whether empathy mediates the game's impact on climate attitudes, policy preferences, and actions (H3b).”
- In amendment #2 of the preregistration plan, we expressed our plan to study the downstream effects of the climate game on vote intention. Yet, in our US survey, we forgot to include that question correctly. Therefore, we don’t report those analyses in this article.
- The original preregistration and their amendments can be found in the following links:

Original: [aspredicted.org/tm56-5wsr.pdf](https://aspredicted.org/tm56-5wsr.pdf); amendment #1: <https://aspredicted.org/mrgq-7jh9.pdf> , amendment #2: [aspredicted.org/ytnr-mrkg.pdf](https://aspredicted.org/ytnr-mrkg.pdf)
